# Pituitary Involvement in Langerhans Cell Histiocytosis: A Challenging Case

**DOI:** 10.7759/cureus.64652

**Published:** 2024-07-16

**Authors:** Aditya Chauhan, Ammar Ahmed, Sreekant Avula, Kimmie Rabe, Allison Estrada

**Affiliations:** 1 Department of Medicine, Division of Endocrinology, Diabetes, and Metabolism, University of Minnesota School of Medicine, Minneapolis, USA; 2 Department of Pathology and Laboratory Medicine, Hennepin County Medical Center, Minneapolis, USA; 3 Department of Endocrinology, Diabetes, and Metabolism, Hennepin County Medical Center, Minneapolis, USA

**Keywords:** pituitary, hypothalamic-pituitary axis, central diabetes insipidus, hypopituitarism, langerhans cell histiocytosis

## Abstract

Langerhans cell histiocytosis (LCH) is a rare disorder involving an abnormal clonal proliferation of precursor cells of the mononuclear phagocytic system. The hypothalamic-pituitary axis is commonly affected by central nervous system (CNS) involvement, with central diabetes insipidus being the most common endocrine abnormality observed. We report the case of a 55-year-old female presenting with vision changes and found to have a hypothalamic mass that was responsive to high-dose steroids. After an initial diagnostic dilemma, the surgical pathology eventually confirmed the diagnosis of LCH. She is being treated with hormone supplementation for panhypopituitarism and intensity-modulated radiation therapy (IMRT) for the LCH. Our case highlights that LCH can present as isolated hypothalamic-pituitary involvement. Early diagnosis is critical to prevent extensive progression of the disease, ultimately leading to permanent physical and endocrine abnormalities. More studies are required to develop specific guidelines and approaches for patients with isolated hypothalamic-pituitary involvement due to LCH.

## Introduction

Sellar and suprasellar neoplasms have a broad differential. Among the comparatively less frequent causes in that list is Langerhans cell histiocytosis (LCH) [1]. LCH is a rare disorder involving an abnormal clonal proliferation of precursor cells of the mononuclear phagocytic system. It has expression of CD1a, CD207, and S100 [2,3]. LCH is predominantly seen in the pediatric population compared to adults [2]. The disease can manifest with single or multiple organ system involvements [2]. The skeletal system, skin, and lungs are commonly involved. The central nervous system (CNS) has been noted to be involved in 3.4-57% of cases [2]. In cases of CNS involvement, the hypothalamic-pituitary axis is commonly affected, and central diabetes insipidus (CDI) is the most common endocrine abnormality seen. Anterior pituitary dysfunction, when present, is mostly permanent, and growth hormone deficiency is the most common hormonal deficiency [2,4]. We present the case of a 55-year-old female with a progressively increasing hypothalamic mass who presented with vision changes and panhypopituitarism with an initial diagnostic dilemma and was found to have LCH.

## Case presentation

Initial encounter

**Figure 1:**
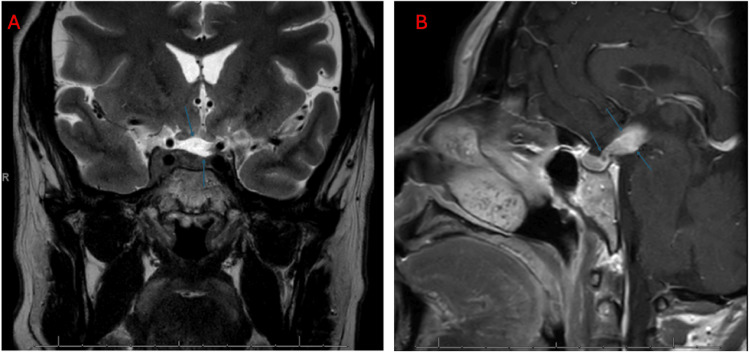
MRI of the brain with and without contrast: coronal (A) and sagittal (B) images demonstrating a heterogeneously enhancing suprasellar mass (blue arrows) measuring approximately 1.2×1.1×1.7 cm involving the optic chiasm and infundibulum with superior extension into the hypothalamus MRI: magnetic resonance imaging

A 55-year-old Hispanic female with no significant past medical history first presented in November 2022 with frontal headaches that had been getting worse over several months and intermittent blurring of her vision; a week before the initial presentation, her symptoms had gotten worse. A computed tomography (CT) of the head without contrast demonstrated a hyperdense lesion along the pituitary infundibulum extending superiorly and posteriorly to the sella turcica and involving the optic chiasm. A magnetic resonance imaging (MRI) of the brain with and without contrast also demonstrated a T2 hyperintense and heterogeneously enhancing mass measuring approximately 1.2×1.1×1.7 cm involving the optic chiasm and infundibulum with superior extension into the hypothalamus. Optic radiations also appeared to be bilaterally involved, as well as proximal pre-chiasmatic optic nerves and the right cavernous sinus (Figure 1).

The hormonal evaluation indicated panhypopituitarism and mild hyperprolactinemia, likely due to the stalk effect. Hormonal levels are presented in Table 1.

**Table 1:** Hormonal assessment during the initial admission and during the admission when the final diagnosis was made ACTH: adrenocorticotropic hormone; FSH: follicle-stimulating hormone; IGF1: insulin-like growth factor 1; TSH: thyroid-stimulating hormone

Tests	Results (initial admission)	Results (admission when the final diagnosis was made)	Normal range
Plasma ACTH (pg/mL)	20.7	2.1	7.2-63.3
Plasma cortisol (mcg/dL)	7.5	0.5	-
Plasma estradiol (pg/mL)	12.6	N/A	-
Plasma FSH (U/L)	2.7	2.6	-
Plasma LH (U/L)	<0.3	<0.3	1.7-8.6
Plasma IGF1 (ng/mL)	52	N/A	51-233
Plasma prolactin (ng/mL)	82.2	63.8	4.8-23.3
Plasma TSH (mIU/L)	4.8	1.42	0.27-4.20
Plasma free T4 (ng/dL)	0.7	0.6	0.9-1.7

A lumbar puncture was performed, and a cerebrospinal fluid (CSF) analysis was unrevealing. Notably, serum IgG4 levels were normal (39.3 mg/dL (2.4-121.0 mg/dL)), and serum angiotensin-converting enzyme (ACE) levels were normal (31 U/L (16-85 U/L)). 

Neurosurgery was consulted, and a biopsy of the pituitary lesion was not pursued as it was deemed to be a high-risk procedure given the involved structure. Medical treatment was recommended at that stage. Despite the mass affecting the optic tracts and producing intermittent blurriness in vision, ophthalmology's official visual field testing revealed that the patient's visual fields were completely intact. According to the neurosurgery team's recommendations, the patient was started on oral dexamethasone 4 mg three times daily with a plan to taper gradually after two weeks to the physiological maintenance dose of oral prednisone 5 mg daily. The patient was started on oral levothyroxine for possible central hypothyroidism. Notably, the electrolytes were within normal limit; however, the patient was polyuric with a low urine osmolarity of 118 mOsm/kg (50-800 mOsm/kg) and a low urine specific gravity of less than 1.005 (1.003-1.030) raising suspicion for diabetes insipidus. Water deprivation testing was not performed as the clinical suspicion was high, and the patient responded clinically to oral desmopressin. 

Interval follow-up

Approximately 20 days after the first MRI, the patient underwent a repeat MRI of the brain with and without contrast. By then, the patient had been taking a high dose of dexamethasone for about 10 days. The suprasellar lesion showed a moderate to significant improvement in its overall size, measuring roughly 9 mm in the axial plane compared to 14 mm on previous imaging and 9.5 mm in the anterior-posterior diameter compared to 12 mm. Additionally, there was an improvement in the cavernous sinus, infundibulum, optic radiations, and optic chiasm involvement. The steroid dose was tapered down to the physiological maintenance dose of prednisone 5 mg daily. A month later, a follow-up brain MRI revealed that the lesion's size had shrunk even more. Around May 2023, the patient started to complain of generalized fatigue and malaise. There were concerns regarding medication compliance. Her levothyroxine dosage was adjusted after the given repeat free T4 level was 0.5 ng/dL (0.8-1.6 ng/dL). A repeat MRI of the brain with and without contrast revealed an interval increase in the pituitary lesion measuring 18×6×5 mm and increasing abnormal T2 signal in the adjacent optic tracts. The patient was subsequently started on oral prednisone 40 mg daily for a month, followed by a taper to prednisone 5 mg daily. Two months following the steroid burst, a follow-up brain MRI with and without contrast showed a reduction in the pituitary lesion size 15×4×4 mm and a decrease in T2 hyperintense signal within the optic chiasm. After a multidisciplinary team discussion, the decision was made to refer her to interventional radiology (IR) to obtain an imaging-guided biopsy of the pituitary lesion which was being planned; however, an additional hospitalization was required beforehand. 

Final diagnosis

**Figure 2:**
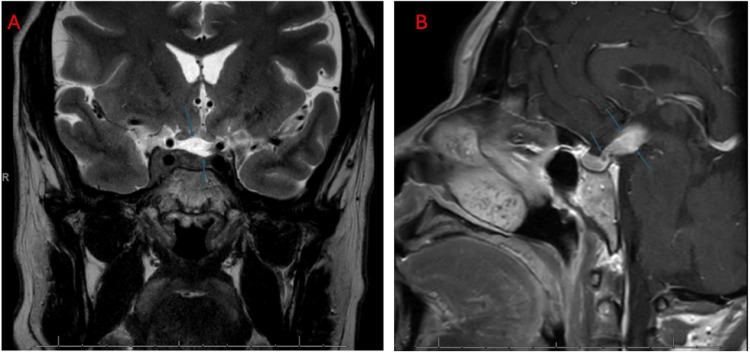
MRI of the brain with and without contrast: coronal (A) and sagittal (B) images demonstrating marked interval enlargement in the suprasellar mass (blue arrows) measuring approximately 3.0×2.6 cm MRI: magnetic resonance imaging

The patient was represented in February 2024 with chief complaints of gradually worsening confusion, intermittent headaches, and decreasing vision in the left eye in the setting of medication non-compliance. Laboratory investigations are summarized in Table 1. Laboratory investigations were notable for serum sodium 151 mmol/L (135-148 mmol/L), 8 AM cortisol 1.66 mcg/dL (6.0-18.4 mcg/dL), ACTH 2.1 pg/mL (7.2-62.3 pg/mL), prolactin 63.8 ng/mL (4.8-23.3 ng/mL), free T4 0.6 ng/dL (0.9-1.7 ng/dL), and TSH 1.42 mIU/L (0.27-4.20 mIU/L). MRI of the brain with and without contrast demonstrated marked enlargement in the suprasellar mass measuring 3.0×2.6 cm with surrounding T2 hyperintensity in the bilateral internal capsules, sublentiform region, midbrain, and optic radiations. Additionally, the third ventricle seemed involved, and the optic chiasm was completely encased (Figure 2).

Diffuse visual field defects on the left, with right temporal field defect, were noted during ophthalmological assessment. The patient subsequently underwent a left parietal craniotomy with partial resection of the tumor. Intraoperatively, a large tumor with a fibrous capsule compressing the optic chiasm was noted. The tumor appeared to be adhered to the optic nerve and the hypothalamus. The left optic nerve was decompressed during the surgery.

**Figure 3:**
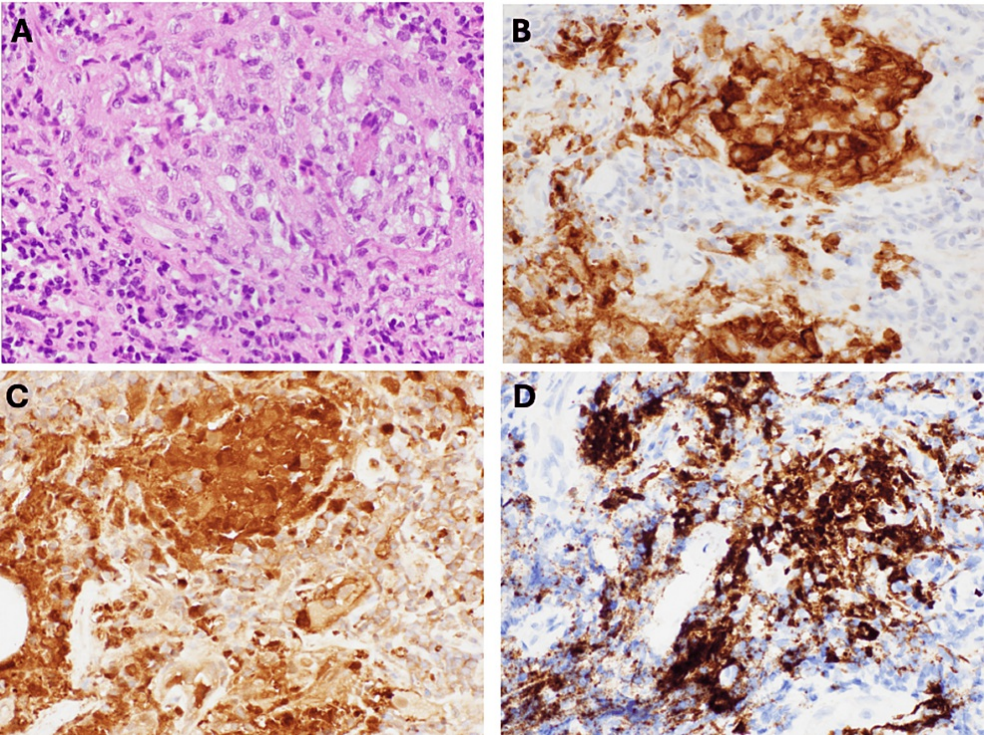
Langerhans cell histiocytosis. The lesions are composed of numerous Langerhans cells and eosinophils (A). The Langerhans cells demonstrate strong membranous and perinuclear staining for CD1a (B). There are nuclear and cytoplasmic staining for S100 (C) and granular and cytoplasmic staining for Langerin (CD207) (D). Hematoxylin-eosin stain (A) and all immunohistochemical stains (B-D) at magnification 400×

The pathology revealed extensive infiltrates, including Langerhans cells, reactive macrophages, lymphocytes, plasma cells, and eosinophils. Immunohistochemistry was positive for CD1a, Langerin, S100, and CD207 (Figure 3).

Immunohistochemistry was negative for BRAF V600E. The final diagnosis was LCH. Further molecular testing via FoundationOne Heme revealed MAP2K1(MEK1)P105_A106del with suggested targeted therapies such as cobimetinib. However, the status of the microsatellite and tumor mutation burden could not be determined. A positron emission tomography (PET)/CT scan did not demonstrate any other areas of involvement. The patient is now following medical oncology and radiation oncology. She has started treatment with intensity-modulated radiation therapy (IMRT). She continues to follow endocrinology for the management of hypopituitarism. The ophthalmology examination conducted two months following the procedure indicates that there has been no additional progression of vision impairment.

## Discussion

LCH is the most prevalent histiocytic illness [3]. Generally, LCH is considered an uncommon condition in adults. In contrast to the pediatric population, the occurrence of this condition in adults is not as clearly characterized and is probably underestimated due to its diverse clinical manifestations [5]. LCH in adults is most seen in males and usually after the fourth decade of life [2,5]. Existing literature indicates that there may be variations in the occurrence of LCH based on ethnicity [5,6]. The occurrence of LCH has been found to be more prevalent in individuals of Hispanic descent, particularly when both parents are of Hispanic origin [6]. Here, we present a case of a middle-aged Hispanic female who had an isolated involvement of the hypothalamic-pituitary region with LCH.

The pathogenesis of LCH involves clonal expansion of myeloid precursors differentiating into CD1a+ and CD207+ cells [2]. Our understanding of molecular pathogenesis has evolved over the years, and it's well established that activation of the mitogen-activated protein kinase (MAPK) pathway plays a critical role [7]. Most cases have activating somatic mutation in BRAF V600E [7]. Other reported mutations include MAP2K1, ARAF, HRAS, NRAS, and ERBB3 [8,9]. The knowledge of these mutations is essential as they carry therapeutic implications. In our patient, BRAF V600E was negative. However, FoundationOne testing found a genomic alteration in MAP2K1(MEK1). MEK inhibitors have a potential role in this type of mutation. 

The clinical presentation of LCH represents a broad spectrum ranging from single-system involvement to disseminated disease [3]. CNS involvement in LCH usually occurs with multisystem disease. It can be in the form of either focal mass lesions or progressive neurodegenerative disease [10,11]. Among the CNS involvement, hypothalamic-pituitary involvement is the most common [12,13]. CDI is the most commonly encountered LCH-associated endocrinopathy, which can present either as an isolated deficiency or in combination with other anterior pituitary hormonal dysfunctions [12,13]. Across studies, the prevalence of CDI has a wide range, with as high as up to 94% in the presence of one or more other pituitary hormonal abnormalities [13-15]. Among anterior pituitary dysfunction, GH deficiency is the most common, with a prevalence between 53% and 67%, and almost always occurs with CDI [13-15]. This is followed by gonadotropin deficiency. Although isolated deficiencies have been reported, secondary hypothyroidism and adrenal insufficiency are generally seen as a part of panhypopituitarism [4,13]. As seen in our case, LCH can also involve the hypothalamus. Apart from causing pituitary dysfunction, hypothalamic involvement can also lead to behavioral or neuropsychological disturbances. Increased appetite, sleep disturbance, and abnormal body temperature regulation are some of the presentations of hypothalamic involvement [4]. Notably, our patient exhibited symptoms such as behavioral alterations, including hyperphagia, at a later stage following radiation treatment.

Nevertheless, it is challenging to determine whether these alterations in behavior are associated with the disease or the radiotherapy treatment, considering that there has been a slight improvement in the lesion size on radiological examination after radiation therapy. Another point that merits discussion is that our patient primarily presented with symptoms resulting from optic chiasm compression by the mass, and it was only during the assessment that she had hypopituitarism. Individual differences may exist in how people perceive certain symptoms as crucial to their overall well-being. However, this is relevant as the lack of early symptoms leads to a delayed diagnosis and causes significant physical impairment, such as visual field defects. This is in contrast to the literature, where patients mostly presented with vague symptoms such as polyuria and polydipsia, indicating endocrine abnormality such as CDI [12,14,16-19].

MRI without contrast and with gadolinium contrast is an ideal imaging method for assessing sellar and suprasellar masses [20]. Typical MRI findings include infundibular thickening and the absence of the "bright spot" of the posterior pituitary, which is especially seen in cases with LCH-induced CDI [14]. Along with the suprasellar mass, involvement of the pons, basal ganglia, and white matter can also be seen [10]. The clinical and radiological findings should complement histopathology for a confirmatory diagnosis. Immunohistochemical positive stains in LCH include CD1a, S100, and (or) CD207 [3]. The MRI, in our case, highlights the aggressive nature of LCH and the importance of early diagnosis. Moreover, our case demonstrates that isolated LCH involving the hypothalamic-pituitary region can be difficult to diagnose. The anatomical location and depth of the hypothalamic-pituitary region in the anterior skull base can make the biopsy difficult and delay the diagnosis. High-dose steroid use before the biopsy can affect the results by suppressing the disease process. 

Treatment of hormonal deficiencies and disease-specific treatment are the two main components of management. The hormonal deficiencies are treated as any other panhypopituitarism [3,13]. The hormonal deficiencies are permanent and usually require lifelong treatment. Regular monitoring needs to be done to identify and treat any new hormonal deficiency [4]. The endocrine management in our patient has been difficult due to medication non-compliance and frequent hospitalizations for behavioral changes. The current standard of treatment in multisystemic LCH includes chemotherapy, such as vinblastine with prednisone [3]. There is also an emerging role of targeted therapies such as BRAF and MEK inhibitors [3]. There is also a limited role of radiotherapy in patients with hypothalamic-pituitary axis involvement [4].

## Conclusions

When approaching a pituitary mass, the differentials should be broad, and LCH should be considered a possibility. The presentation of LCH represents a broad spectrum. Our case highlights that LCH can present as isolated hypothalamic-pituitary involvement. Due to the vague initial clinical symptoms, the diagnosis can be delayed. Early diagnosis is critical to prevent extensive progression of the disease, ultimately leading to permanent physical and endocrine abnormalities. More studies are required to develop specific guidelines and approaches for patients with isolated hypothalamic-pituitary involvement due to LCH.
